# Pooled CRISPR screening in pancreatic cancer cells implicates co-repressor complexes as a cause of multiple drug resistance via regulation of epithelial-to-mesenchymal transition

**DOI:** 10.1186/s12885-021-08388-1

**Published:** 2021-05-29

**Authors:** Ryne C. Ramaker, Andrew A. Hardigan, Emily R. Gordon, Carter A. Wright, Richard M. Myers, Sara J. Cooper

**Affiliations:** 1 University of Alabama-Birmingham, Birmingham, AL 35294 USA; 2 HudsonAlpha Institute for Biotechnology, Huntsville, AL 35806 USA; 3 University of Alabama – Huntsville, Huntsville, AL 35899 USA

**Keywords:** Genome-wide screen, CRISPR, Pancreatic cancer, Drug resistance, ABCG2, HDAC1, Precision oncology

## Abstract

**Background:**

Pancreatic ductal adenocarcinoma (PDAC) patients suffer poor outcomes, including a five-year survival of below 10%. Poor outcomes result in part from therapeutic resistance that limits the impact of cytotoxic first-line therapy. Novel therapeutic approaches are needed, but currently no targeted therapies exist to treat PDAC.

**Methods:**

To assess cellular resistance mechanisms common to four cytotoxic chemotherapies (gemcitabine, 5-fluorouracil, irinotecan, and oxaliplatin) used to treat PDAC patients, we performed four genome-wide CRISPR activation (CRISPR_act_) and CRISPR knock-out (CRISPR_ko_) screens in two common PDAC cell lines (Panc-1 and BxPC3). We used pathway analysis to identify gene sets enriched among our hits and conducted RNA-sequencing and chromatin immunoprecipitation-sequencing (ChIP-seq) to characterize top hits from our screen. We used scratch assays to assess changes in cellular migration with HDAC1 overexpression.

**Results:**

Our data revealed activation of ABCG2*,* a well-described efflux pump, as the most consistent mediator of resistance in each of our screens. CRISPR-mediated activation of genes involved in transcriptional co-repressor complexes also conferred resistance to multiple drugs. Expression of many of these genes, including HDAC1, is associated with reduced survival in PDAC patients. Up-regulation of HDAC1 in vitro increased promoter occupancy and expression of several genes involved in the epithelial-to-mesenchymal transition (EMT). These cells also displayed phenotypic changes in cellular migration consistent with activation of the EMT pathway. The expression changes resulting from HDAC1 activation were also observed with activation of several other co-repressor complex members. Finally, we developed a publicly available analysis tool, PancDS, which integrates gene expression profiles with our screen results to predict drug sensitivity in resected PDAC tumors and cell lines.

**Conclusion:**

Our results provide a comprehensive resource for identifying cellular mechanisms of drug resistance in PDAC, mechanistically implicate HDAC1, and co-repressor complex members broadly, in multi-drug resistance, and provide an analytical tool for predicting treatment response in PDAC tumors and cell lines.

**Supplementary Information:**

The online version contains supplementary material available at 10.1186/s12885-021-08388-1.

## Background

Despite decades of clinical trials evaluating dozens of potential therapeutics, pancreatic ductal adenocarcinoma (PDAC) has remained largely refractory to improvement of five-year survival rates, which are still less than 10% [1, 2]. With largely non-specific symptoms and invasive procedures required for diagnosis, only 20% of PDAC patients are eligible for surgical resection, leaving a majority of patients with chemotherapy and radiation as their sole course of treatment [1]. Even patients eligible for surgical resection tend to have recurrent disease within 2 years at which time the cancer typically is refractory to adjuvant chemotherapy [3]. Multi-drug combinations, such as FOLFIRINOX (fluorouracil, folinic acid, irinotecan and oxaliplatin), achieve, at best, modest improvements in patient outcomes over single-agent treatment [4]. Unfortunately, many people with pancreatic cancer develop complete resistance to potent multi-drug cocktails [2].

The limited effectiveness of cytotoxic chemotherapy has led to a long effort to identify alternative treatment strategies. Large cohort sequencing efforts have established a relatively short list of commonly mutated genes, including *KRAS*, *TP53*, *SMAD4*, and *CDKN2A*, however these have thus far proven to be poor drug targets [5–7]. Moreover, few genetic perturbations have been reproducibly associated with patient prognosis [7, 8]. An alternative strategy is to develop therapies that reverse resistance or increase sensitivity to existing treatments.

Cellular mechanisms of resistance have been explored by previous insertional mutagenesis- and RNA interference-based screens and have successfully identified genes whose inactivation leads to gemcitabine sensitivity in PDAC cells [9–11]. Genome-wide CRISPR-Cas9 screening has been shown to provide complementary information to these previously developed genetic screening methods [12, 13] and, pertinent to PDAC, has identified essential genes, including FZD5, in cell lines with *RNF43*-mutations (a recurrently mutated gene in PDAC) [14]. These prior studies suggest genome-wide screens have the potential to identify novel mechanisms of chemoresistance, but comprehensive screening for mechanisms of resistance to multi-drug cocktails commonly used to treat PDAC patients is currently lacking [15].

**Fig. 1:**
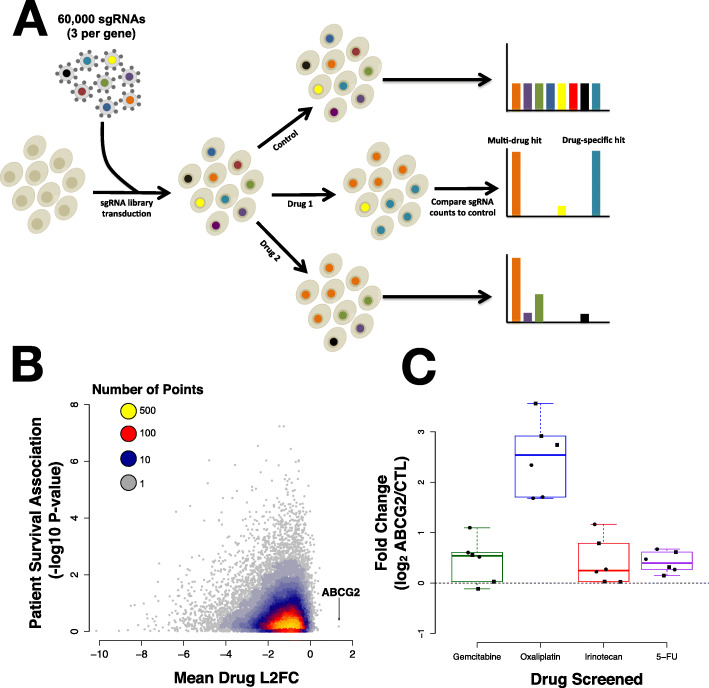
CRISPR screen reveals drug resistance genes. **A** Schematic describing our screening protocol. **B** A scatterplot shows the mean L2FC sum for all four drugs assayed in each of two cell lines compared to the log_10_ *p*-value for the association of the same gene’s expression with with patient survival. ABCG2 stands out as the most highest L2FC over all four drugs. **C** Boxplots indicate the sgRNA fold change in counts per million comparing treated cells to control cells for each replicate and each cell line for the top *ABCG2* sgRNA. Circles represent data from Panc-1 cells. Squares represent BXPC-3

The growing field of precision oncology aims to predict an optimal treatment for a patient based on genomic profiling. One group has begun to look for gene expression signatures associated with drug response in patient-derived PDAC organoids [16]. However, in the context of highly heterogeneous tumors, detection of genetic signatures associated with treatment response is difficult [17]. One tractable approach to this problem is to define the landscape of cellular mechanisms of PDAC drug resistance experimentally, then deeply screen tumors in a targeted manner for the presence of previously-identified resistance drivers. To achieve this goal, we performed CRISPR-Cas9 knock-out (CRISPR_ko_) [18] and endogenous activation (CRISPR_act_) [19] screening of 23,728 genes using 138,188 sgRNAs in two PDAC cell lines (BxPC3 and Panc-1) to identify genes whose loss or gain of expression were able to modulate sensitivity to four common cytotoxic chemotherapies used in the treatment of PDAC (gemcitabine, oxaliplatin, irinotecan, and 5-fluorouracil, Fig. 1A, S1A-B). We identified both known and novel resistance genes and further investigated how HDAC1 overexpression regulates epithelial-to-mesenchymal transition (EMT) leading to a stem-like phenotype and drug resistance.

## Methods

### Reagents

#### Cell culture

Panc-1 (CRL-1469), BxPC3 (CRL-1687), and MiaPaca-2 (CRL-1420) cell lines were obtained from ATCC. All cell lines were maintained in DMEM (ThermoFisher #11965) supplemented with 10% FBS (VWR #16777), 0.5% Penicillin-Streptomycin (ThermoFisher #15140122), and 0.5% GlutaMAX (ThermoFisher #A12860). All cell lines were maintained at 37 °C and 5% CO_2_. HEK 293FT cells (ThermoFisher #70007) were cultured in DMEM (ThermoFisher #11965) supplemented with 10% FBS (VWR #16777), 0.5% Penicillin-Streptomycin (ThermoFisher #15140122), 0.5% Non-essential amino acids (ThermoFisher #11140), and 0.5% sodium pyruvate (ThermoFisher #11360070).

#### Plasmids

LentiCas9-Blast (Addgene #52962) or Lenti-dCAS9-VP46-Blast (Addgene #61425) and lenti-MS2-p65-HSF1-Hygro (Addgene #61426) were used to generate stable cell lines for gene knockout and activation, respectively. We used the GeCKO A pooled sgRNA library (Addgene #1000000049) and the SAM pooled sgRNA library (Addgene #1000000057) for gene knockout and activation screening. We used lentiSAMv2 (Addgene #75112) for single gene activation validation. Guides were cloned as described previously [20]. pMD2.G (Addgene #12259) and psPAX2 (Addgene # 12260) were used to facilitate viral packaging of sgRNA pools and single vector plasmids.

#### Library amplification

The GeCKO A and SAM library were amplified as described previously (20). Briefly, for each pooled library 8 electroporations were performed using 1uL of library and 25uL of Lucigen Endura Electrocompetent cells (Lucigen #60242). Pooled transformation were plated on 825cm^2^ bioassay plates with LB agar containing ampicillin and grown for 14 h at 35 °C. Transformation efficiency was greater than 1 × 10^8^ for both libraries. Bacterial colonies were collected, pelleted and stored at -20 °C for less than 1 week. Plasmid DNA was extracted from greater than 3 g of pellet using the Qiagen EndoFree Plasmid Maxi Kit (Qiagen #12362).

Primer sequences used for amplification are available in Supplemental Table S1**.**

#### Viral packaging

HEK293T cells were grown to 60% confluency in 225cm^2^ flasks. To proceed with viral packaging, DMEM was removed and replaced with 13 mL of pre-warmed OptiMEM (ThermoFisher #31985). The pMD2.G, psPAX2, and pooled library plasmids were combined with Lipofectamine PLUS reagent (ThermoFisher #15338) and OptiMEM. The combined solution was added and incubated for 6 h, when OptiMEM was replaced with media supplemented with 1% bovine serum albumin (ThermoFisher #A9647). After 60 h, media was harvested, centrifuged at 3000 rpm for 10 min at 4 °C, filtered through a 0.45um filter (VWR #28145), and stored at 4 °C overnight in Lenti-X concentrator (Clontech #631231) at a 3:1 virus to Lenti-X concentrator volume ratio. The following day the virus was centrifuged at 1500×g for 45 min at 4 °C and the supernatant was poured off. The viral pellet was resuspended in DMEM culture media at one-tenth the original volume and stored at -80 °C. Packaging of all vectors was performed identically to the pooled library, with reagents scaled down proportionally according to the surface area of the flask.

Viral packaging of single sgRNAs used for validation was performed in 6-well tissue culture treated plates. The protocol was performed as above except cells were 90% confluent, the incubation after transfection was reduced to 48 h and virus was either frozen as before, or in some cases used fresh.

#### Drug resistance screening

Panc-1 and BxPC3 cell lines were transduced with LentiCas9-Blast or co-transduced with Lenti-dCAS9-VP46-Blast and lenti-MS2-p65-HSF1-Hygro for knock out and activation screening, respectively. 500,000 cells were seeded into each well of a six-well flask with 0.8μg/mL polybrene (Sigma #TR-1003-G). Previously packaged plasmid was added at volumes that ranged from 0 to 500uL of virus. Cells were then centrifuged at 2000 rpm for 2 h at 37 °C. The following day, Panc-1 cells were treated 10μg/mL Blasticidin and 1500μg/mL Hygromycin and BxPC3 cells were treated with 2.5μg/mL Blasticidin and 1250μg/mL Hygromycin for 1 week until control cells (no virus added) were dead. The lowest viral titer that had a sufficient number of cells to carry forward were grown up such that pooled libraries could be transduced at 500x representation at a multiplicity of infection (MOI) of 0.4.

Pooled library transduction was performed in at least 30 wells of a 12 well flask at 3 × 10^6^ cells per well with 0.8μg/mL polybrene and sufficient volume of concentrated virus to reach an MOI of 0.4 (approximately 30% of cells surviving after antibiotic selection). Cells were transduced as described above. The following day cells were split into larger 225cm^2^ flasks and either puromycin (Panc-1 10μg/mL, BxPC3 2.5μg/uL) or zeocin (Panc-1 2 mg/mL, BxPC3 3 mg/mL) was added to select for presence of knock out and activation plasmids, respectively. Library-transduced cells were under selection for 1 week post-transduction and expanded to 7 × 10^7^ cells per treatment replicate or 1000x representation for each of the 4 drugs (gemcitabine, oxaliplatin, irinotecan and 5-fluorouracil) and control conditions per replicate. Drug concentrations were as follows for Panc-1 cells: Gemcitabine (25 nM) Oxaliplatin (2.5uM), Irinotecan (500 nM), 5-FU (7.5uM) and for BxPC3: Gemcitabine (25 nM), Oxaliplatin (2.5uM), irinotecan (250 nM), 5-FU (5uM). A minimum 500x representation was maintained at all times in control cells. Drug treatment doses were optimized to yield ~ 80% cell death relative to untreated control cells after 14 days of culture. After 14 days of drug treatment, cells were pelleted and stored at -80 °C. DNA extraction and library preparation was performed as previously described [20].

To identify top genes from our genome-wide screen, we prioritized genes by the “L2FC sum” in each cell line, which is the sum of the replicate minimum log_2_ fold changes of the top two sgRNAs targeting each gene. Multi-drug hits were prioritized by computing the mean “L2FC sum” of the four drug treatments.

#### Single gene validation

The top sgRNAs for genes of interest were cloned into either LentiCrispr-v2 or LentiSAMv2 for knock-out or activation screen validation as described previously [19]. (Sequences are available in Supplemental Table S2). To generate stable cell lines expressing each sgRNA, single sgRNA plasmids underwent viral packaging and were transduced. For transduction, 10^6^ cells were seeded per well of a 6-well plate for a total volume of 2 mL of cells and media. To that we added, 1.6ul of polybrene (Millipore Sigma #TR-1003-G), and 1 mL of packaged virus. Then, we used spinfection for 30 min at 2000RPM at 37C. All lines were established using antibiotic selection as described above.

Non-targeting and targeting sgRNAs were plated in 96-well plates at 750 cells/well and treated with a range of gemcitabine, oxaliplatin, or irinotecan concentrations. Plated cells were grown for 6 and 8 days, for the knockout and activation screen, respectively, with the media changed and drug applied every 2 days. The number of viable cells surviving drug treatment was assayed with CellTiter-Glo (Promega #G7571). ABCG2 inhibition was done under these conditions as well, with the only change being the addition of 3uM sorafenib or 3uM KO143 where indicated.

#### Sequencing and data processing for drug resistance screen

Three sets of replicates (control and 4 drug treated samples) for each cell line were sequenced on one lane of Illumina NextSeq resulting in an average of 40 million reads per sample. 5′ and 3′ adapters were observed in > 99% of reads and were trimmed using cutadapt [21]. Adapter trimmed fastq files were then aligned to the sgRNA libraries and raw count tables generated using MAGeCK [22]. We had a perfect alignment rate of 72–74% of raw reads for each sample. Sequencing the control samples revealed sufficient representation of guides with an average of 99.8 and 99.3% of sgRNAs detected and 98.4 and 94.2% of sgRNAs detected at greater than 1 read per million in our knock out and activation control samples, respectively. A log_2_ fold change was computed for each sgRNA in each drug treated sample relative to the untreated control sample for each replicate. sgRNAs with fewer than 10 counts in the untreated control samples and fewer than 50 counts in a treated control sample were excluded from further analysis (< 1% of sgRNAs). At this step we recognized the first replicate of our knock-out screen correlated relatively poorly with the second two replicates and was excluded from downstream analysis. We ranked each of the sgRNAs targeting each gene by the minimum log_2_ fold change across each replicate. Top genes were subsequently prioritized for follow up by their “L2FC sum” in each cell line, which is the sum of the replicate minimum log_2_ fold changes of the top two sgRNAs targeting each gene (Supplemental Table S3A-B). Multi-drug hits were prioritized by computing the mean “L2FC sum” across each of the four drug treatments.

#### Pathway analysis

Pathways enriched for genes conferring drug resistance were identified by comparing the distribution of log_2_ fold changes for the top, second, and third sgRNA targeting each gene within a Reactome pathway to that of all other genes by Wilcox ranked sum test. Reactome [23] pathways with fewer than 10 genes targeted in our libraries were excluded. A consolidated knock out and activation score was computed for each pathway by summing the –log_10_ *P*-values for the top, second, and third sgRNAs of each pathway (Supplemental Table S4).

#### Predicting drug response using genome-wide screening results

Sensitivity to each of the four drugs was computed using a cell line’s treatment naïve gene expression levels and the minimum L2FC Sum for each gene across both cell lines. Raw expression data for each cell line, obtained from sources described below, was processed to obtain raw count tables [24]. Variance stabilizing transformation was used to normalized expression and a z-score was calculated [25]. Next, a scalar was computed to weight each gene’s expression level using the L2FC Sum. We computed the weighted expression values for each gene. Finally, the cumulative sum was calculated to generate a single value representing a cell line’s expected level of resistance to the given drug. Irinotecan sensitivity and gene expression data were obtained for 18 PDAC cell lines with permission from the Cancer Cell Line Encyclopedia [26]. (https://portals.broadinstitute.org/ccle). Gemcitabine sensitivity (cell lines were classified as sensitive, intermediate, or resistant) and gene expression data for 14 PDAC cell lines was obtained from previously published work [27]. A panel of five PDAC cell lines was screened for oxaliplatin sensitivity by treating with serial dilutions of the drug as described previously [27]. Cell counts at each dose were compared to a vehicle control to construct a six-point dose response curve. The area under the dose-response curve was used to compare sensitivity. Patient expression data and treatment info was obtained from a previous study using the GEO accession GSE79670.

#### Survival analysis

To create an integrated analysis of our screen results (knockout and activation) and patient overall survival, we downloaded survival statistics for 185 PDAC patients who participated in the TCGA study (https://gdac.broadinstitute.org/). We calculated a cox proportional hazard ratio and associated *p*-value DESeq2 VST normalized transcripts using the R ‘Survival’ package ‘coxph’ function.

#### Genomic analysis of HDAC1 and repressor gene over-expressing lines

Cell lines over-expressing chromatin remodelers, including HDAC1 were characterized using RNA-sequencing (RNA-seq) and ChIP-sequencing (ChIP-seq). Data were generated and analyzed using established, published methods [28, 29]. Briefly, RNA-sequencing libraries for MiaPaca-2 and Panc-1 cells were made using the Lexogen 3′ RNA-sequencing kit. They were pooled and sequenced on an Illumina NextSeq instrument with 75 bp single-end reads. Analysis used previously published methods including: Trimgalore for adapter trimming, fastqc for quality score assessment, STAR for alignment to hg38, HTSeq-gene to map reads to features, and DESeq2 for differential expression analysis. For ChIP-seq, HDAC1 over-expressing MiaPaca-2 cells were cross-linked, harvested, and DNA was precipitated using a commercial HDAC1 antibody (Invitrogen, PA1860). Libraries were constructed, pooled and sequenced using Illumina NovaSeq single end 75 bp reads. These data were generated and analyzed using published ENCODE protocols (https://www.encodeproject.org/documents/).

#### Scratch assays

MiaPaca-2 cells over-expressing ABCG2 or expressing a non-targeting guide were used for scratch assays. Scratch assays were completed using standard growth conditions after plating 1 × 10^5^ cells. Cell migration was assessed using images captured every 8 h for 64 h on a Lionheart FX (Biomek). Based on merged images collected over 64 h, we calculated the time (hr) to close the gap by half the width as previously described [30]. Images were compiled in R (version 3.5.0) and analyzed with GIMP (v. 2.10) and Image J (version 1.5.2q).

#### Data availability

Raw sequencing data from our screen are available at SRA by referencing the BioProject number PRJNA542321.

RNA-sequencing and ChIP-sequencing data are available using the GEO accessions GSE131596 (ABCG2 only) and GSE158541 (all other data).

## Results

We performed a genome-wide knock out and activation screen to identify genes that conferred resistance to each of four chemotherapy drugs used in the treatment of PDAC: gemcitabine, irinotecan, 5-fluorouracil, and oxaliplatin. We calculated enrichment for individual sgRNAs in either the activation or knock out screen to identify genes associated with drug resistance. We found the sgRNAs most strongly associated with drug resistance were highly drug-specific (Figure S1B). Replicate samples were significantly more correlated than samples treated with a different drug (Figure S1C-F). However, there was a much higher correlation between samples treated with different drugs than expected by chance, suggesting that mechanisms of resistance were sometimes shared between drugs in our study (Figure S1C-F). To prioritize resistance genes, we computed the sum of the replicate-minimum, log_2_ count fold change (L2FC) of the two most enriched sgRNAs targeting each gene in each cell line. We identified multi-drug resistance genes by computing the mean L2FC sum across all four drugs in each cell line (All data: Supplemental Table 3A-B, Top Hits by cell line and drug: Supplemental Table 4A-B). This approach was particularly powerful because it leveraged information from 48 perturbations (4 drug screens × 3 replicates × 2 cell lines × 2 sgRNAs).

In the CRISPRko screen, we observe minimal overlap between cell lines and drugs. We calculated a combined L2FC Sum representing the combined results of all cell lines and drugs aimed at identifying multi-drug resistance genes. None of these signals reached statistical significance. We also identified overlapping genes from the 5-FU and gemcitabine screens. Since these drugs are both nucleoside analogs, we anticipated we might see similar hits. Again, the overlap was minimal but among the top hits in both screens was the gene VPS25, which is a tumor suppressor known to inhibit the Notch pathway [31]. Inhibition of VPS25 in model systems induces proliferation and reduced expression is associated with modestly reduced survival in the TCGA cohort (*p* = 0.17). Based on these results, we focused our analysis on the activation screen, which revealed more genome-wide significant hits and an integrated analysis of the two screens described below.

**Fig. 2:**
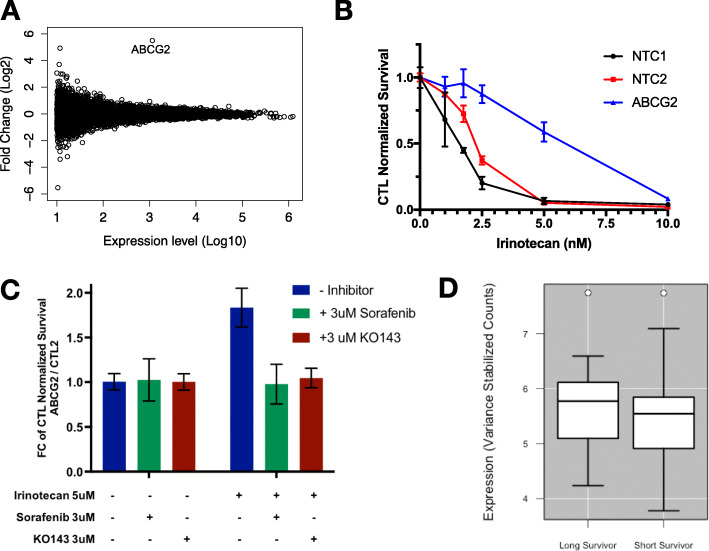
**A** Scatterplot shows ABCG2 stands out as the single gene showing a highly significant change in expression with use of the ABCG2_A and ABCG2_B sgRNAs to activate ABCG2 expression. These data are generated from RNA-sequencing of MiaPaca-2 and Panc-1 cells over-expressing ABCG2. **B** Cell survival curves display the fraction of cells surviving after a series of irinotecan doses for Panc-1 cells stably expressing an *ABCG2*-targeting sgRNA (blue) or non-targeting control sgRNAs (red and black). **C** Each bar represents a ratio of ABCG2 over-expressing cells compared to a non-targeting control with the indicated drug treatments (+ indicates a treatment was added). Treatment of MiaPaca2 cells over-expressing ABCG2 using the ABCG2 guide shows no significant cell death with inhibitor only, where as ABCG2 alone confers drug resistance. The combination of 3uM sorafenib (green) or KO143 (maroon) with ABCG2 overexpression restores normal sensitivity to Irinotecan. **D** Boxplots showing the normalized expression levels of *ABCG2* in Kirby et al. for patients surviving < 300 days or > 900 days. *P*-value is not significant

Analysis of the activation screen data revealed a common resistance gene. CRISPR_act_ of the ATP-binding cassette (ABC) transporter, ABCG2, was the only perturbation that persistently induced resistance across each of the drug treatments in both of our cell lines **(**Fig. 1B-C). We created a stable Panc-1 cell line over-expressing ABCG2 using our top ABCG2 sgRNA. RNA-sequencing showed a highly specific, 30-fold overexpression of ABCG2 (Fig. 2A). These cells also showed a strong resistance phenotype (Fig. 2B). Similar resistance was observed in other pancreatic cancer lines, BxPC-3 and MiaPaca-2, with ABCG2 overexpression (Supplemental Figure S2A-B). We treated MiaPaca-2 cells over-expressing ABCG2 with known inhibitors of the ABCG2 protein, KO149 and Sorafanib, to show that this resistance was reversible (Fig. 2C). While ABCG2 over-expressing cells show resistance to irinotecan as measured by the ratio of the number of ABCG2 over-expressing cells to control treated cells, treatment with either inhibitor eliminates the growth advantage provided by ABCG2. ABCG2 functions as an efflux pump with a broad range of substrates and has been associated with multi-drug resistance in several previous studies [32–34]; these results provide a strong validation of the efficacy of our multi-drug screening approach. Despite being the strongest signal associated with multi-drug resistance in our screen, ABCG2 expression does not appear to have clinical relevance in PDAC patient tumors, as it is expressed at relatively low levels in all prognosis groups (Fig. 2D).

**Fig. 3:**
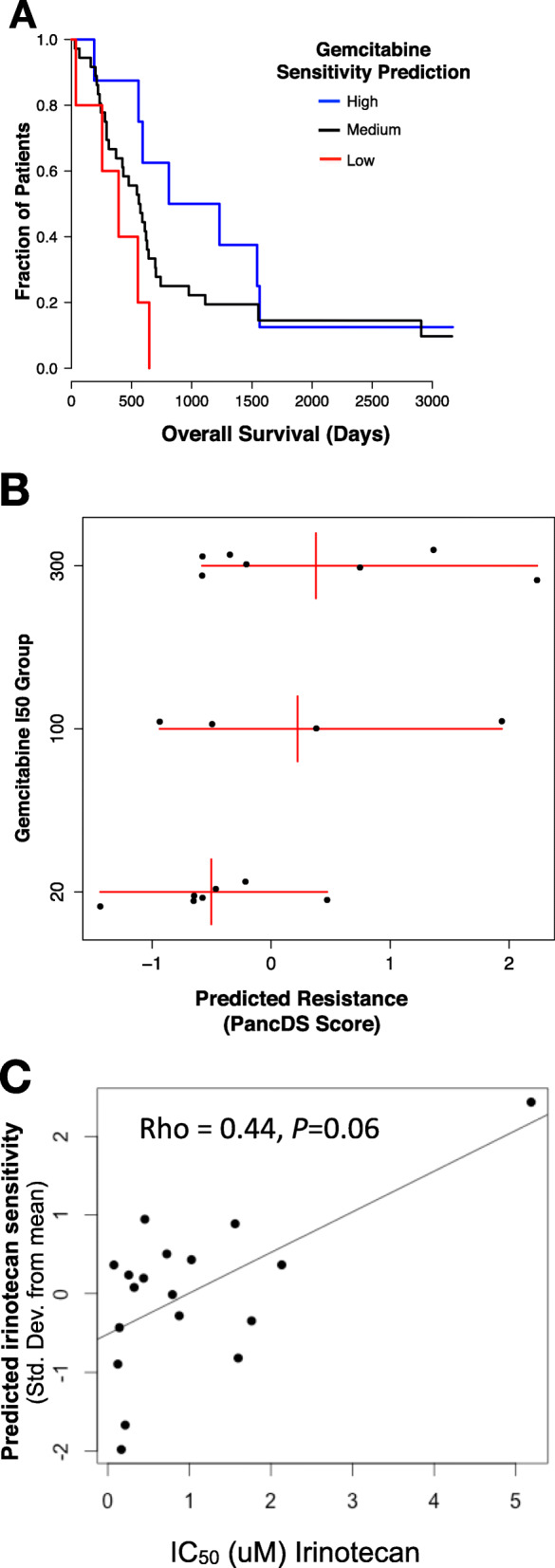
**A** Using weighted averages derived from the screen data we predicted likely sensitivity to gemcitabine based on expression of resistance-associated genes (PKG). There is a significant difference in survival between patients with predicted high versus low gemcitabine sensitivity (*p* = 0.01, Chi-squared test). **B** Predicted Gemcitabine Sensitivity was calculated using a weighted average of gene expression for resistance-associated genes based on expression profiles for 18 pancreatic cancer cell lines. These data were plotted compared to experimentally measured IC_50_ groups (Low: IC_50_ < 20uM, Moderate: 100uM < IC_50_ < 300; High: IC50 > 300uM. The Wilcoxon *P*-value between the most resistant group of cell lines and the most sensitive is 0.097. **C** Scatterplot showing observed irinotecan IC_50_ values compared to predicted sensitivity for 18 PDAC cells assayed by the Cancer Cell Line Encyclopedia. (Rho = 0.44, *p* = 0.06)

To assess the clinical relevance of our screening data, we developed an analytical approach that uses our results to predict the sensitivity of treatment-naïve PDAC tumors and cell lines to our assayed drugs. We reasoned that if drug resistance genes identified in vitro are relevant in patients, we might be able to use expression of these genes to predict patient outcomes. We computed predicted drug sensitivity scores based on a weighted expression level of resistance genes included in our screen using the algorithm we developed and have made freely available, PancDS (https://github.com/rramaker/PancDS). This script computes predicted drug sensitivity scores from gene expression data. We computed gemcitabine sensitivity scores and successfully stratified gemcitabine-treated PDAC patients into groups with distinct overall survival (Fig. 3A). We also applied this algorithm to predict response to gemcitabine and irinotecan treatment in a panel of PDAC cell lines (Fig. 3B-C).

**Fig. 4:**
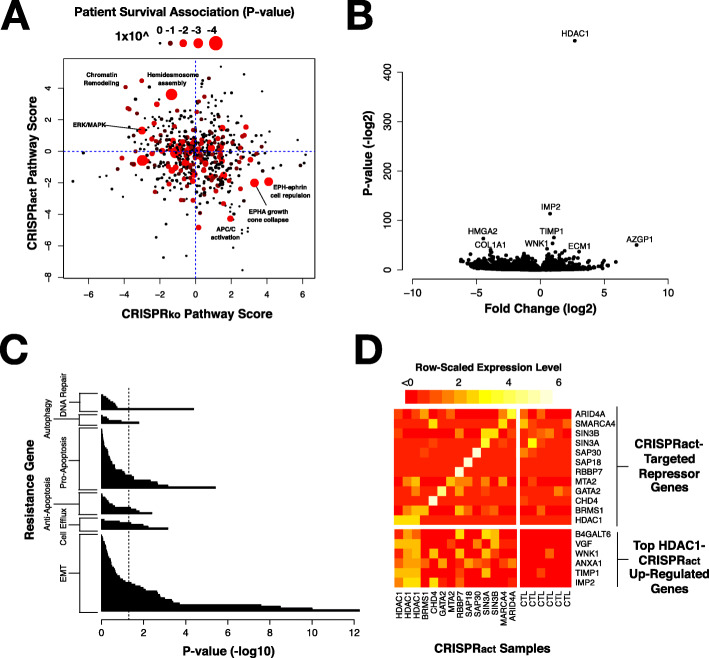
**A** Scatter plot showing pathways enriched for multi-drug resistance in our CRISPR_act_ and CRISPR_ko_ screens and their association with patient survival in the TCGA cohort. Patient survival is represented by the size and color of the circle. **B** A volcano plot shows that activation of HDAC1 expression using a dCas9-activation approach results in strong overexpression of HDAC1 based on RNA-sequencing. **C** Pathway enrichment analysis shows that HDAC1 overexpression especially affects epithelial-to-mesenchymal transition (EMT), cell efflux, apoptosis, autophagy, and DNA repair. *P*-values reported are derived from Fisher’s exact test comparing observed versus expected number of genes in each pathway. A full list is available in Supplemental Table S5. **D** Overexpression of each of the target genes listed on top right quadrant leads to a similar pattern of overexpression of genes shown on the lower right

In addition to the utility of these data for predicting treatment response, they also can be used to uncover individual mechanisms of multi-drug resistance with clinical relevance. We investigated gene pathways that were over-represented among the genes that exhibited drug resistance or sensitivity based on our CRISPR_act_ and CRISPR_ko_ screens (Supplemental Table S5). We tested whether expression of genes in those pathway were associated with patient survival. We identified several pathways that met this criteria including chromatin remodeling, hemidesmosome assembly, and ERK/MAPK signaling (Fig. 4A). These pathways, which when activated induce drug resistance and when knocked out confer drug sensitivity, represent potentially useful therapeutic targets.

While several of these pathways are of interest, we focused on activation of chromatin remodeling genes as one of the consistent mechanisms of drug resistance. The role of chromatin remodeling in pancreatic cancer has been heavily investigated as several genes involved in histone methylation (*MLL2* and *MLL3*) and members of the tumor suppressing SWI/SNF complex (*SMARCA1* and *ARID1A*) are recurrently mutated in PDAC tumors [35]. Moreover, global changes in repressive histone marks has been associated with metastatic PDAC tumors [36]. We found that activation of several genes involved in the repression of chromatin via histone deacetylation resulted multi-drug resistance (Supplemental Figure S3). Particularly prominent were members of the Nucleosome Remodeling Deacetylase (NuRD) complex (*MTA2/3*, *CHD3/4*, *HDAC1/2,* and *GATAD2A*), members of the Nuclear receptor CoRepressor (NCoR) complex (*NCOR2*, *TBL1XR1*, and *TBL1X*), and the repressive transcription factor *REST* and its interacting partner *RCOR1*, each of which have been demonstrated to regulate gene expression programs important for chemoresistance [37–39]. Widespread chromatin repression has also been previously associated with poor prognosis in PDAC patients [36]. Notably, histone deacetylases are key members of each of these three complexes and were also among the highly ranked genes in our activation screen. Based on these data, we performed targeted CRISPR_act_ experiments in Panc-1 and MiaPaca-2 cells with HDAC1, which cooperates with trans-acting repressors as a member of several transcriptional repressor complexes. Our top HDAC1 sgRNA induced greater than 10-fold overexpression of HDAC1. Overexpression of this transcriptional regulator also produced several weaker transcriptional changes that we hypothesize to be downstream of HDAC1-mediated transcriptional regulation (Fig. 4B). We sought to understand how these myriad gene expression changes might be linked to multi-drug resistance. Previous studies have implicated DNA repair, autophagy, apoptosis, drug efflux and the epithelial-to-mesenchymal transition (EMT) in drug resistance [40, 41]. An analysis of genes in each of these pathways shows that gene expression changes resulting from HDAC1 overexpression were particularly enriched for genes implicated in the epithelial-to-mesenchymal transition (EMT), a pathway known to mediate multi-drug resistance (Fig. 4B-C) [35]. Given HDAC1’s function as a member of canonical repressor complexes, we were surprised to find that its activation also resulted in several up-regulated genes, including IMP2, TIMP1, ANXA1, and WNK1, which are involved in promoting a mesenchymal or stem cell state [42–45]. IMP2 was the most up-regulated gene we observed upon HDAC1 overexpression. It drives a less differentiated state via activation of Notch signaling, a known mechanism of drug resistance in pancreatic cancer [46, 47]. While expression of many EMT associated genes is altered with HDAC1 expression, we did not observe altered expression of canonical EMT transcriptional regulators Snail (SNAI1), Slug (SNAI2) or Twist (TWIST1).

Our pathway analysis of the screen data indicated that many transcriptional repressors were associated with resistance. In addition to analysis of HDAC1 overexpression, we used CRISPR_act_ to stably over-express 11 other transcriptional repressors (ARID4A, SMARCA4, SIN3A, SIN3B, SAP30, SAP18, RBBP7, MTA2, GATA2, CHD4, and BRMS1) and assay gene expression in these cell lines using RNA-seq. These lines showed concordant up-regulation of at least one, but often several, of the same top genes over-expressed upon HDAC1-activation (Fig. 4D).

**Fig. 5:**
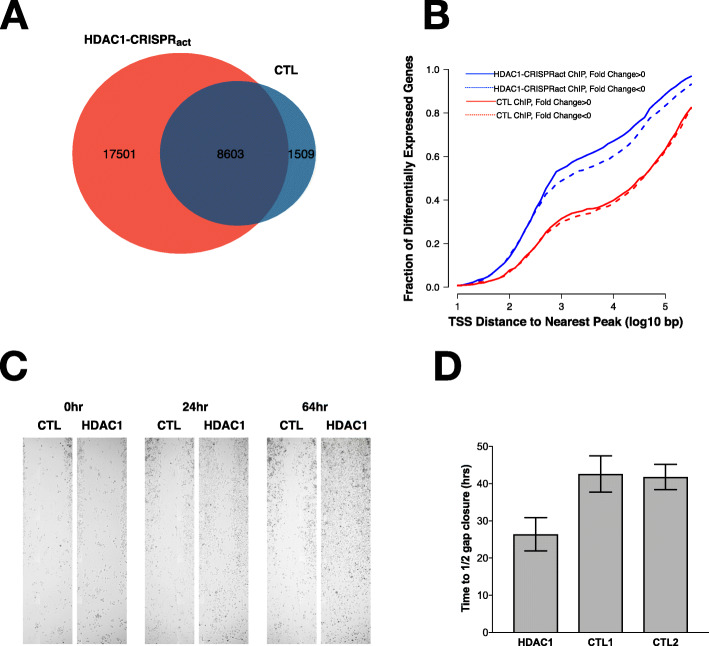
**A** ChIP-seq analysis reveals ChIP-seq peaks in the control (CTL) MiaPaCa-2 cells overlap significantly with MiaPaCa-2 cells over-expressing HDAC1 (red). An additional 17,501 peaks are identified with overexpression of HDAC1. **B** Cumulative distribution plot showing that HDAC1 binding sites identified upon overexpression of HDAC1 (blue) are nearby transcription start sites (TSS) of differentially expressed genes. **C** Scratch assay shows that overexpression of HDAC1 leads to increased migration compared to control cells. **D** Quantification of the scratch assays shows a significant difference with HDAC1 overexpression (Student’s T-test)

We wanted to better understand how HDAC1 overexpression might lead to drug resistance via induction of EMT. We used ChIP-sequencing (ChIP-seq) to identify HDAC1 binding sites in cells with activated HDAC1 compared to the wild-type parent MiaPaca-2 cell line to further understand the impacts of HDAC1 overexpression. We observed a 3-fold increase in the number of sites occupied with HDAC1 after overexpression relative to control cells and identified 17,501 occupied sites specific to HDAC1 activation (Fig. 5A). These new HDAC1 ChIP-seq peaks represent additional binding sites for HDAC1 with overexpression and were highly enriched near the transcription start sites of genes differentially expressed upon HDAC1 activation (Fig. 5B), suggesting a role for direct binding of HDAC1 in modulating their expression. Specifically we observed that HDAC1 binds the promoters of key EMT genes including WNK1, TIMP1, and IMP2/IGF2BP2. WNK1 and IMP2 both show increased occupancy at the HDAC1 overexpression lines compared to the controls (Supplemental Figure S4). Finally, we tested the functional impact of HDAC1 activation on cells. Using a scratch assay, we found that HDAC1-activated cells exhibited significantly increased cell migration relative to control cells as measured by the time required to close the width of the gap by half (Fig. 5C-D).

## Discussion

Large-scale screening efforts that both inhibit and endogenously activate expression of genes have revolutionized forward genetic screen and are empowering discoveries that were previously not possible [48]. Here, we have presented a large screen to reveal known and novel mechanisms of chemoresistance in PDAC, the first screen to perform both knock out and activation in PDAC cells. The large size of the screen allowed us to recognize some general features of drug resistance screens using CRISPR_ko_ and CRISPR_act_. We observed a relatively weak correlation between replicate reproducibility and gene significance levels across cell lines. While this observation may be partially explained by methodological covariates like selection strength, it is also likely that strong genetic and phenotypic differences observed between these cell lines permit different mechanisms of chemoresistance [49]. A gene capable of modulating chemosensitivity across every PDAC cell line would be an ideal target, however, our data suggests that this is not a realistic expectation. Many previous screens for chemoresistance in PDAC cell lines have been limited in that they only use one cell line [9, 50] and our study likely under samples the natural variation in PDAC with only two cell lines. As sequencing and screening costs decrease, future studies examining orders of magnitude greater numbers of cell lines will likely add to the knowledge catalogued by our study.

While large-scale screening efforts like this one represent an important step in the fight to reverse resistance and improve treatment options for PDAC, our study is also limited in that it identifies only cellular mechanisms of resistance. These data should be considered only part of the picture since the tumor microenvironment also plays a critical role in chemoresistance [51].

The data we generated demonstrate the value of pooled CRISPR screening as a method for discovering cellular mechanisms of drug resistance. CRISPR_act_ of *ABCG2* was the highest-confidence perturbation capable of inducing multi-drug resistance. We confirmed ABCG2 overexpression in three pancreatic cancer lines resulted in resistance to chemotherapy and we showed that chemical inhibition of ABCG2 can reverse resistance in vitro, but its clinical relevance is unclear given a lack of strong correlation with patient outcomes. Some reports indicate that *ABCG2* is expressed uniquely in cancer stem cells, a minority of the total cell population; thus, *ABCG2’*s role may be obscured in bulk tumor sequencing [34].

While ABCG2 expression is not strongly linked to patient outcomes, we showed that overall, hits from our screen are relevant to patient outcomes. Moreover, we leveraged our screen data to predict drug sensitivity in cell lines and to predict outcomes in patients based on their respective gene expression profiles demonstrating the potential application of these data, en masse, to direct personalized therapeutic approaches.

Our pathway-based analysis linked CRISPR_act_ of several transcriptional repressor complex members to chemoresistance. Another recent screen also implicated HDAC Class I signaling in resistance to dasatinib, a tyrosine kinase inhibitor is being tested in PDAC cell line [52]. We observed that overexpression of several chromatin remodeling genes, including *HDAC1*, induced a program of gene expression associated with epithelial-to-mesenchymal transition (EMT). EMT has been associated with drug resistance for several decades but the mechanisms by which EMT is induced vary between cancers and cell lines [41, 53–55]. The EMT pathway has been previously associated with chemoresistance in mouse models of PDAC where key EMT transcription factors were ablated [55]. Acquisition of EMT phenotype has previously been associated with gemcitabine resistance in pancreatic cancer cell lines [46]. These authors showed that activation of Notch signaling was required. Our data suggests that activation of Notch signaling can also be achieved through chromatin remodeling, and perhaps driven by overexpression of IMP2. Chromatin remodeling genes have also been linked to clinically relevant phenotypes in PDAC [56] including induction of EMT and increasing stem cell populations [57, 58]. We noted that while dozens of genes associated with EMT were differentially regulated upon overexpression of HDAC1, some key regulators known to drive EMT like Snail, Slug, Twist, and Zeb1 were not significantly differentially expressed [59, 60]. Our HDAC1 ChIP-seq data integrated with RNA-sequencing from HDAC1 overexpressing lines demonstrates that *HDAC1* overexpression significantly increases occupation of HDAC1 at the promoter regions of genes important for the regulation of EMT and those changes likely drive chemoresistance. It is worth noting however that we observe a broad reprogramming that includes altered expression of genes involved in cell proliferation, migration and adhesion (all components of EMT) as well as the regulation of apoptosis.

An interesting observation from our integration of HDAC1 binding and gene expression was that despite its canonical role as a repressor, HDAC1 was capable of binding and impacting transcription of its targets both as a repressor and as an activator. Recent studies have observed that HDAC complexes can play a variety of roles in gene regulation [61]. In fact, some of the key genes involved in EMT (e.g. TIMP1, WNK1, and IMP2) were up-regulated following the overexpression of HDAC1.

Given the link we observed between HDAC1 overexpression and chemoresistance, it follows logically that HDAC inhibition might be a relevant therapeutic strategy. In fact, HDAC inhibitors have exhibited inconsistent results in clinical trials to date, an observation that has been partially attributed to a poor understanding of which HDAC classes are the best targets and which biomarkers indicate sensitivity to HDAC inhibition [62]. For that reason, a deeper understanding of the downstream effectors of resistance following HDAC1 overexpression is critical to identifying alternative and hopefully more suitable therapeutic targets.

## Conclusions

Our screens represent a significant resource in the understanding of chemoresistance in pancreatic cancer. The ability to predict patient outcomes based on the results of this screen speaks to the value of the data for both mechanistic understanding and for clinical application. We have explored one significant pathway resulting from this work to further our understanding of HDAC1 specifically and chromatin remodeling more generally with our data pointing to the relevance of chromatin remodeling for the regulation of the EMT program, which contributes drug resistance. The results of our large-scale screen provide additional avenues for exploration in the important quest to improve treatment options for pancreatic cancer patients.

## Supplementary Information

**Additional file 1: Supplemental Table S1.** Primer Sequences for sgRNA Library Construction. **Supplemental Table S2.** sgRNA sequences for CRISPRact of individual genes and controls. **Supplemental Table S3A.** Activation (SAM) Screen results. L2FC Sum for individual drug-cell line combinations and combined. **Supplemental Table S3B.** Knockout (Gecko) Screen results. L2FC Sum for individual drug-cell line combinations. **Supplemental Table S4A.** Top 10 hits for each drug in each cell line for knockout (GecKO) Screen. **Supplemental Table S4B.** Top 10 hits for each drug in each cell line for Activation (SAM) Screen. **Supplemental Table S5.** Results of pathway analysis and Survival *p*-values used to make Fig. 2A.

**Additional file 2: Figure S1.** (A) Schematic detailing the screening workflow. (B) A heatmap of the sgRNA count per million, z-scored by row for the most variable 500 sgRNAs across all of our CRISPRa screen replicates in the PANC-1 cell line. (C-F) Boxplots describing the replicate, non-replicate, and scrambled null permutation Spearman correlations for knockout screens in Panc-1 (C) and BxPC3 (E) and activation screens in Panc-1 (D) and BxPC3 (F). **Figure S2**. Irinotecan resistance following overexpression of ABCG2 compared to controls in two cell lines. A) BxPC-3 cells over expressing ABCG2 show resistance to irinotecan (*p* = 0.037, t-test comparing IC50 values for ABCG2 v. NTC2). B) MiaPaca-2 cells over expressing ABCG2 show resistance to irinotecan (*p* = 0.026, t-test comparing IC50 values for ABCG2 v. NTC2). **Figure S3.** The percentile rank for genes known to be involved in chromatin remodeling. HDAC1 is among the genes in this pathway. **Figure S4.** ChIP-seq analysis of HDAC1-overexpressing (red) MiaPaca-2 cells compared to non-targeting controls (blue). HDAC1 overexpressing cells show increased HDAC1 peak height at (A) the IGF2BP2 promoter, (B) the WNK1 promoter, and (C) the B4GALT6 promoter.

## Data Availability

ABCG2 RNA-sequencing data are available at GEO using the accessions GSE131596. RNA-sequencing and ChIP-sequencing data for chromatin remodelers are available using the GEO accession GSE158541.Raw sequencing data from the screen are available through SRA using the project ID PRJNA542321.
