# Supplementary material for: Pooled CRISPR screening in pancreatic cancer cells implicates co-repressor complexes as a cause of multiple drug resistance via regulation of epithelial-to-mesenchymal transition
Source: BMC Cancer. 2021 May 29;21:632. doi: 10.1186/s12885-021-08388-1 (PMC8164247; doi:10.1186/s12885-021-08388-1)
Supplement: Supplementary file 2 — Additional file 2: Figure S1. (A) Schematic detailing the screening workflow. (B) A heatmap of the sgRNA count per million, z-scored by row for the most variable 500 sgRNAs across all of our CRISPRa screen replicates in the PANC-1 cell line. (C-F) Boxplots describing the replicate, non-replicate, and scrambled null permutation Spearman correlations for knockout screens in Panc-1 (C) and BxPC3 (E) and activation screens in Panc-1 (D) and BxPC3 (F). Figure S2. Irinotecan resistance following overexpression of ABCG2 compared to controls in two cell lines. A) BxPC-3 cells over expressing ABCG2 show resistance to irinotecan (p = 0.037, t-test comparing IC50 values for ABCG2 v. NTC2). B) MiaPaca-2 cells over expressing ABCG2 show resistance to irinotecan (p = 0.026, t-test comparing IC50 values for ABCG2 v. NTC2). Figure S3. The percentile rank for genes known to be involved in chromatin remodeling. HDAC1 is among the genes in this pathway. Figure S4. ChIP-seq analysis of HDAC1-overexpressing (red) MiaPaca-2 cells compared to non-targeting controls (blue). HDAC1 overexpressing cells show increased HDAC1 peak height at (A) the IGF2BP2 promoter, (B) the WNK1 promoter, and (C) the B4GALT6 promoter. [file 12885_2021_8388_MOESM2_ESM.pdf]

Pooled CRISPR screening in pancreatic cancer cells implicates co-repressor complexes as a cause of multiple drug resistance via regulation of epithelial-to-mesenchymal transition

Author list:

Ryne C. Ramaker*[1,2]

Andrew A. Hardigan*[1,2]

Emily R. Gordon*[2]

Carter A. Wright[2,3]

Richard M. Myers[2]

Sara J. Cooper[2]

Supplemental Figure S1

Supplemental Figure S2

Supplemental Figure S3

Supplemental Figure S4

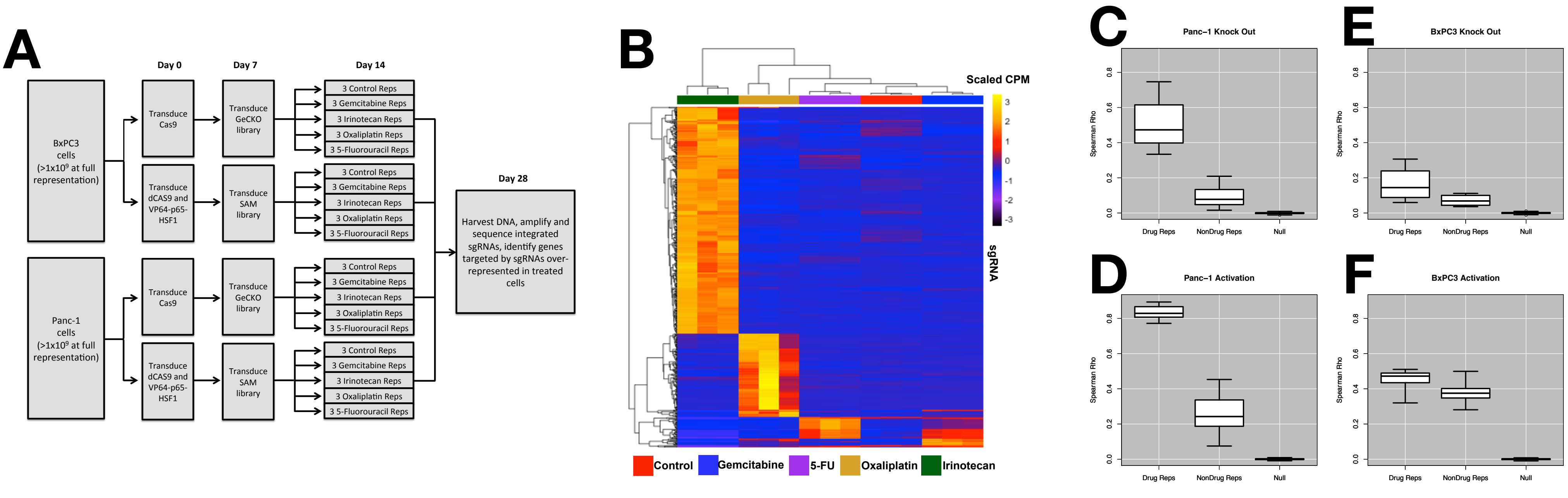

Figure S1. (A) Schematic detailing the screening workflow. (B) A heatmap of the sgRNA count per million, z-scored by row for the most variable 500 sgRNAs across all of our CRISPRa screen replicates in the PANC-1 cell line. (C-F) Boxplots describing the replicate, non-replicate, and scrambled null permutation Spearman correlations for knockout screens in Panc-1 (C) and BxPC3 (E) and activation screens in Panc-1 (D) and BxPC3 (F).

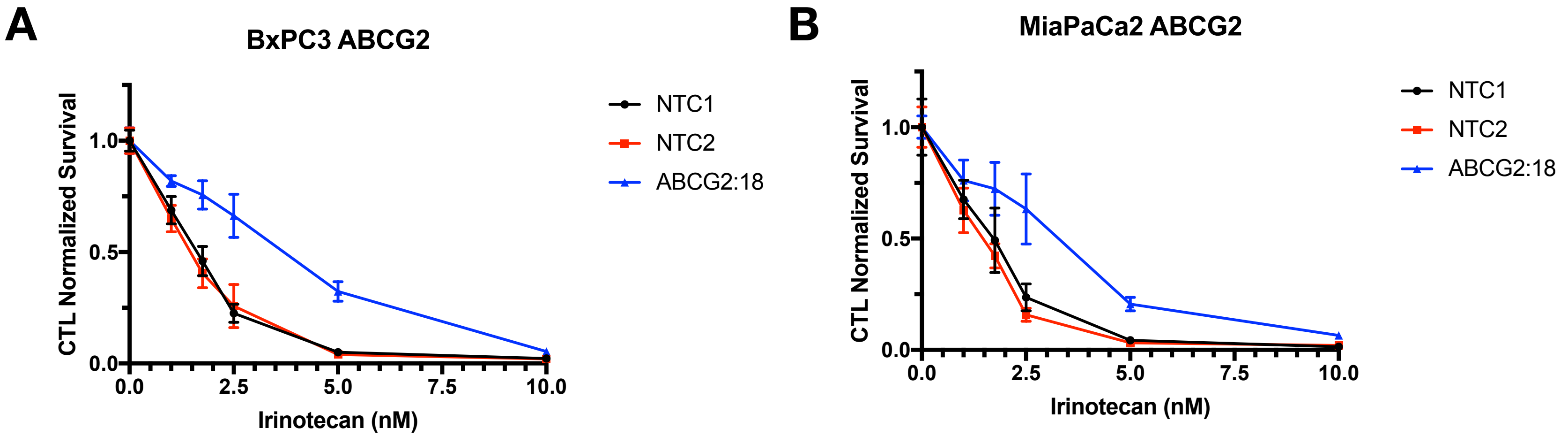

Figure S2. Irinotecan resistance following over-expression of ABCG2 compared to controls in two cell lines. A) BxPC-3 cells over expressing ABCG2 show resistance to irinotecan ($p=0.037$, t-test comparing IC50 values for ABCG2 v. NTC2). B) MiaPaca-2 cells over expressing ABCG2 show resistance to irinotecan ($p=0.026$, t-test comparing IC50 values for ABCG2 v. NTC2)

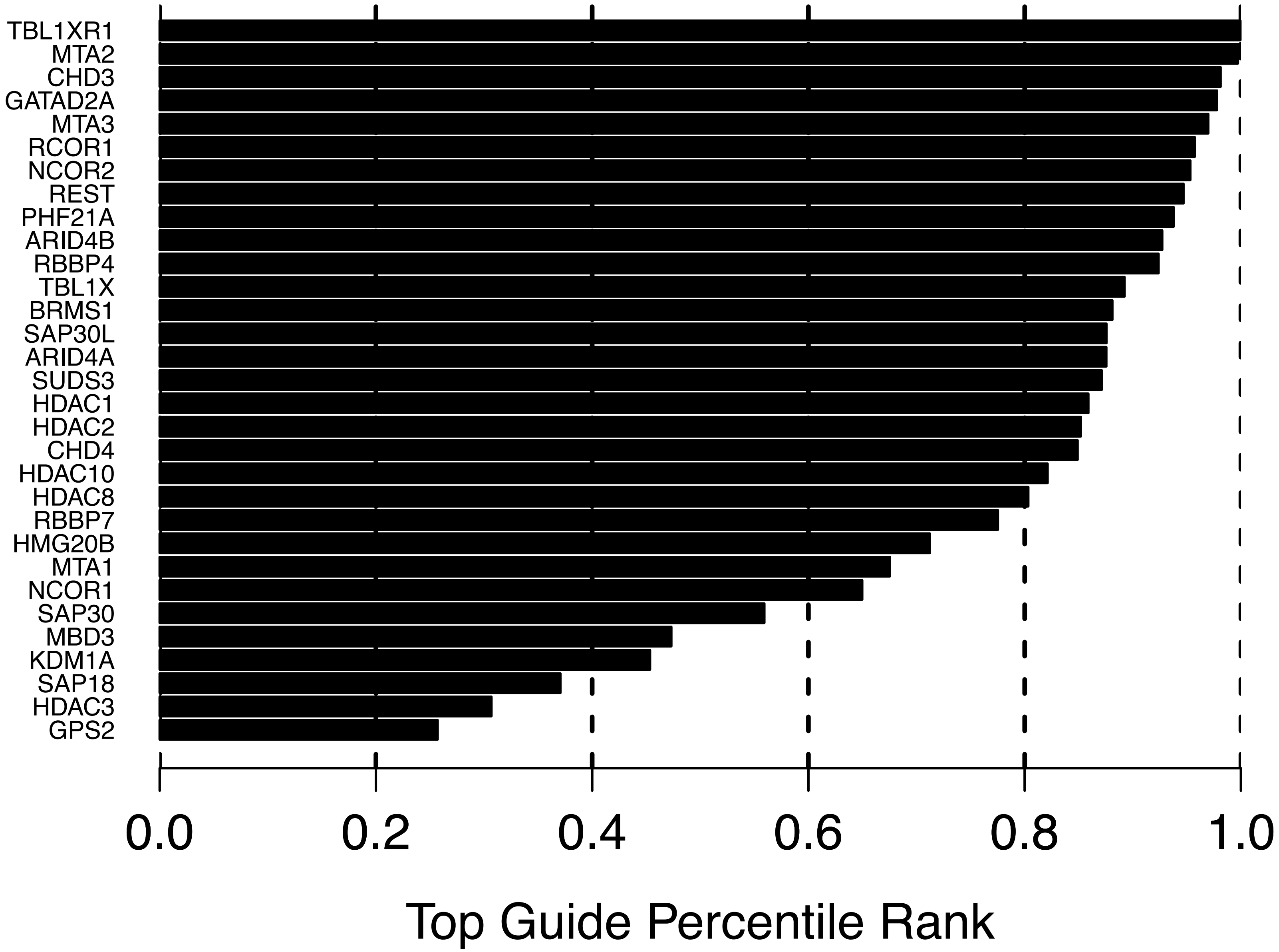

Figure S3. The percentile rank for genes known to be involved in chromatin remodeling. HDAC1 is among the genes in this pathway.

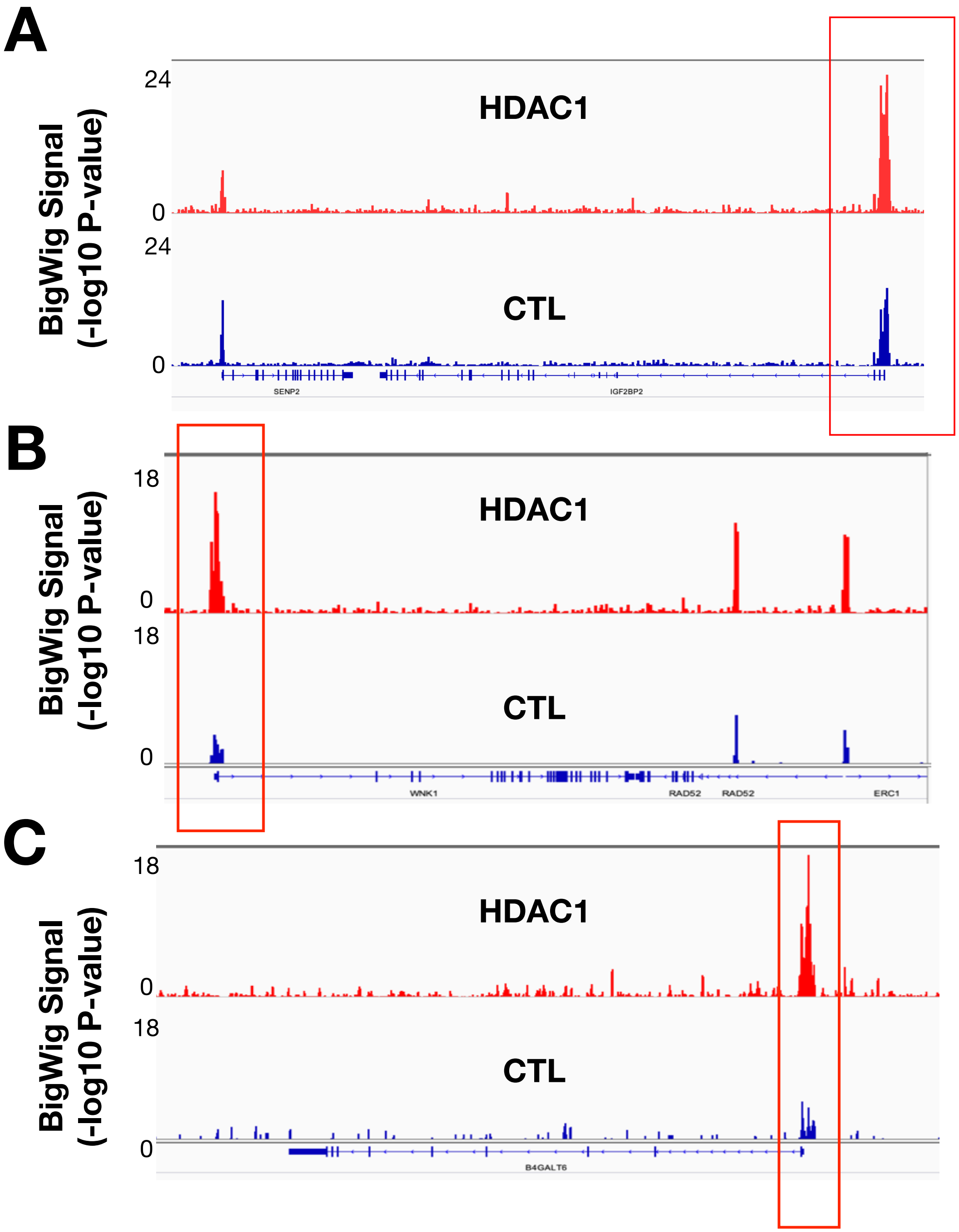

Figure S4. ChIP-seq analysis of HDAC1-overexpressing (red) MiaPaca-2 cells compared to non-targeting controls (blue). HDAC1 overexpressing cells show increased HDAC1 peak height at (A) the IGF2BP2 promoter, (B) the WNK1 promoter, and (C) the B4GALT6 promoter.
